# The role of germline BRCA1 & BRCA2 mutations in familial pancreatic cancer: A systematic review and meta-analysis

**DOI:** 10.1371/journal.pone.0299276

**Published:** 2024-05-29

**Authors:** Edward Kurnia Setiawan Limijadi, Muflihatul Muniroh, Yan Wisnu Prajoko, Kevin Christian Tjandra, Danendra Rakha Putra Respati

**Affiliations:** 1 Doctoral Study Program of Medical and Health Science, Universitas Diponegoro, Semarang, Indonesia; 2 Faculty of Medicine, Department of Clinical Pathology, Universitas Diopnegoro, Semarang, Indonesia; 3 Faculty of Medicine, Department of Physiology, Universitas Diponegoro, Semarang, Indonesia; 4 Faculty of Medicine, Department of Surgical Oncology, Universitas Diponegoro, Semarang, Indonesia; 5 Kariadi General Hospital, Semarang, Indonesia; 6 Faculty of Medicine, Departement of Medicine, Universitas Diponegoro, Semarang, Indonesia; CNR, ITALY

## Abstract

**Background:**

Familial Pancreatic Cancer (FPC) presents a notable risk, with 3–10% of pancreatic adenocarcinoma cases having a family history. Studies link FPC to syndromes like HBOC, suggesting BRCA1/BRCA2 mutations play a role. BRCA gene functions in DNA repair impact FPC management, influencing sensitivity to therapies like PARP inhibitors. Identifying mutations not only aids FPC treatment but also reveals broader cancer risks. However, challenges persist in selectively applying genetic testing due to cost constraints. This Systematic Review focuses on BRCA1/BRCA2 significance in FPC, diagnostic criteria, prognostic value, and limitations.

**Method:**

Original articles published from 2013 to January 2023 were sourced from databases such as Scopus, PubMed, ProQuest, and ScienceDirect. Inclusion criteria comprised observational cohort or diagnostic studies related to the role of BRCA1/2 mutation in correlation to familial pancreatic cancer (FPC), while article reviews, narrative reviews, and non-relevant content were excluded. The assessment of bias used ROBINS-I, and the results were organized using PICOS criteria in a Google spreadsheet table. The systematic review adhered to the PRISMA 2020 checklist.

**Result:**

We analyzed 9 diagnostic studies encompassing 1325 families and 4267 patients from Italy, USA, and Poland. Despite the limitation of limited homogenous PICO studies, our findings effectively present evidence. BRCA1/2 demonstrates benefits in detecting first-degree relatives FPC involvement with 2.26–10 times higher risk. These mutation findings also play an important role since with the BRCA1/2 targeted therapy, Poly-ADP Ribose Polymerase inhibitors (PARP) may give better outcomes of FPC treatment. Analysis of BRCA1 and BRCA2 administration’s impact on odds ratio (OR) based on six and five studies respectively. BRCA1 exhibited non-significant effects (OR = 1.26, P = 0.51), while BRCA2 showed significance (OR = 1.68, P = 0.04). No heterogeneity observed, indicating consistent results. Further research on BRCA1 is warranted.

**Conclusion:**

Detecting the BRCA1/2 mutation gene offers numerous advantages, particularly in its correlation with FPC. For diagnostic and prognostic purposes, testing is strongly recommended for first-degree relatives, who face a significantly higher risk (2.26–10 times) of being affected. Additionally, FPC patients with identified BRCA1/2 mutations exhibit a more favorable prognosis compared to the non-mutated population. This is attributed to the availability of targeted BRCA1/2 therapy, which maximizes treatment outcomes.

## 1. Introduction

Familial Pancreatic Cancer (FPC) is one of the various types of cancer that indicates a family history can be a risk factor for cancer occurrence in other family members. Approximately 3% to 10% of individuals with pancreatic adenocarcinoma have a positive family history related to pancreatic cancer, and it is estimated that about 10% to 20% of pancreatic adenocarcinoma cases are considered to originate from hereditary factors [1,2]. In recognizing these risk factors, several studies have demonstrated the involvement of first-degree relatives (FDR) in pancreatic cancer as well as related syndromes such as Hereditary Breast and Ovarian Cancer (HBOC), Peutz-Jeghers Syndrome (PJs), Lynch Syndrome (LS), and Familial Atypical Multiple Melanoma (FAMM) in the development of cancer in subsequent generations [3–6]. Among these syndromes, HBOC is reported to be closely associated with the occurrence of FPC. This implies that mutations in BRCA1 and BRCA2, commonly found in HBOC, play a role in the development of FPC [7,8].

The function of the BRCA genes is focused on repairing double-stranded breaks (DSBs) in DNA molecules using the homologous recombination (HR) mechanism. Identifying BRCA mutations is a crucial step in the management of prevention, diagnosis, and treatment of FPC. Previous studies have demonstrated that BRCA damage in FPC disrupts the Homologous Recombination pathway, leading to increased sensitivity to PARP inhibitors and platinum-based chemotherapy [8–10]. Furthermore, pancreatic cancer patients who exhibit deficiencies in DNA mismatch repair, as seen in Lynch syndrome, often show a positive response to immune checkpoint inhibitors. Pembrolizumab, one of the drugs that functions as an immune checkpoint inhibitor, has received specific approval for use in patients with tumors that are microsatellite instability-high [11,12].

Besides influencing treatment options, the identification of these mutations is also responsible for providing insights into the risk of other cancers that may be associated with the mutation or syndrome. In some situations, preventive actions through surgery or chemoprevention may also be explained as options. However, genetic testing cannot be applied to all pancreatic cancer patients due to cost considerations and limited overall results. Therefore, a significant challenge in clinical practice remains in selecting FPC patients most suitable for BRCA1/BRCA2 testing or other genetic tests. In this effort, we write this Systematic Review to summarize BRCA1/BRCA2’s significance in FPC, its diagnostic criteria, prognostic value, and its limitations. In addition, to determine whether BRCA1/2 gene mutations are associated with pancreatic cancer in patients with close familial relationships compared to other selection criteria.

## 2. Methods

### Registration and criteria

The Preferred Reporting Items for Systematic Reviews and Narrative Reviews were utilized in this systematic review. On August 8, 2023, it was registered on the Open Science Framework (OSF). Covering the period from 2013 to 2023 (with the final search conducted on September 20, 2023), the study included original research publications that met specific clinical observational criteria, encompassing retrospective cohorts and autologous clinical case-control studies. Various types of publications, such as technical reports, editor responses, narrative reviews, systematic reviews, narrative reviews, non-comparative studies, in silico studies, in vitro studies, in vivo studies, applied scientific posters, research proposals, and conference abstracts, were excluded. Articles not in English, those with incomplete content, and those unrelated to BRCA mutations correlated with FPC were also excluded. The PICO criteria for the selected products focused on the prevalence of BRCA mutations (BRCA1, BRCA2, or BRCA1/2) in male or female FPC patients. Other inclusion criteria considered germline mutations (if specified) and any mutations, irrespective of their founder status.

### Data sources and search strategy

Research was gathered through various databases, including Scopus, PubMed, ProQuest, and Science Direct. These searches covered the period from the establishment of the databases until December 20th, 2023, spanning the preceding 10 years before the review. The Boolean operator was applied to Medical Subject Headings (MeSH) keywords obtained from the National Institute of Health (NIH) National Library of Medicine browser. The search strategy employed the following keywords: "BRCA," "Familial," "Hereditary," and "Pancreatic Cancer". The studies were organized and managed using the Mendeley Group Reference Manager in the authors’ library. Filtering criteria included using clinical-type articles for the PubMed database and research article types for the Scopus, ProQuest, Sage Pub, Cochrane, and Science Direct databases. The database search yielded a total of 3600 studies (588 from Scopus, 1274 from PubMed, 1570 from ProQuest, and 168 from Science Direct. Following the addition of the specified criteria, 1429 studies were imported into the Mendeley Group Reference Manager in the authors’ library, and this occurred before the selection process.

### Selection process

Two independent reviewers (KCT, DRPR) and one validator (EKSL) combined the outcomes from four databases. After that, they conducted a complete text and abstract screening to exclude non-observational papers while keeping the pertinent ones. Several 1413 studies were eliminated from this process because they did not provide sufficient data to answer the research question and because their study designs did not meet the criteria for inclusion (clinical observational) and 7 studies were removed due to duplication. The retrieval of the complete text for the remaining 9 studies was then tested. Only 11 full-text articles could be obtained as a result. Using the Cochrane ROBINS-I tool, the 11 included studies were evaluated for eligibility. All 11 of the listed studies from this process passed the evaluation bias check. The PRISMA flow chart contained records of the research selection procedures.

### Data extraction

After the final screening, a data extraction form was conducted on Microsoft Excel, covering the following information: author, publication year, publishing country, study period, study design, sample size, cancer diagnosis, diagnostic criteria, BRCA pathogenic variants (PVs), other PVs, prevalence, and how outcomes were defined. The data were extracted by two reviewers (KC, DRPR), and disagreements between them were resolved through discussion and input from a validator (EKSL).

### Study risk of bias assessment

The risk-of-bias assessment tool employed to evaluate the bias in the included studies was the ROBINS-I, accessible at (https://methods.cochrane.org/bias/resources/rob-2-revised-cochrane-risk-bias-tool-randomized-trials), and it was utilized by four reviewers (KCT, DRPR, MM, YWP) and one validators (EKSL). Any discrepancies in the bias assessment were thoroughly discussed and resolved among the five reviewers. Papers identified with a high risk of bias will be excluded from the systematic review to maintain the integrity of the included data in the current study, as indicated.

## 3. Results

### Study selection

**Fig 1:**
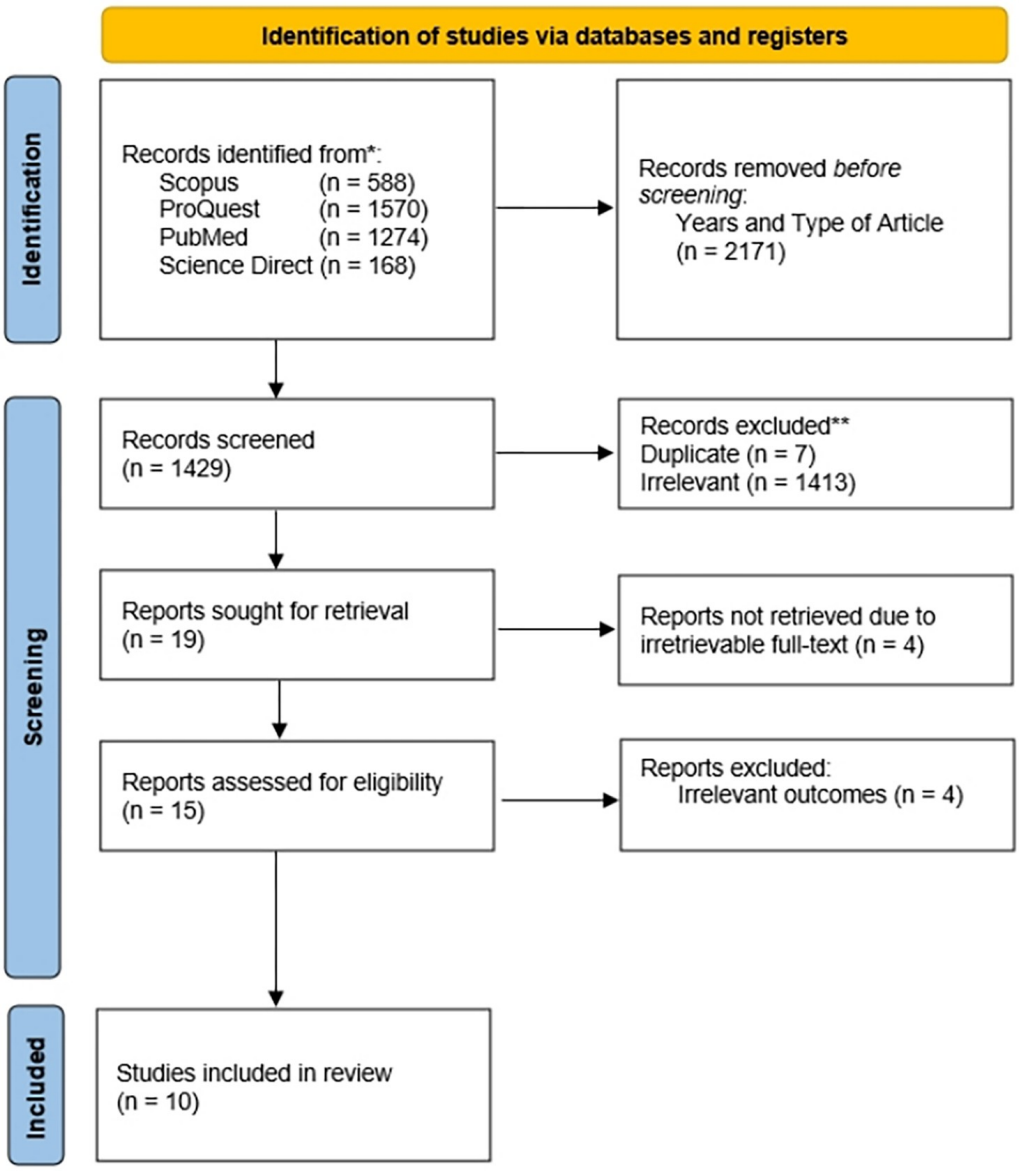
PRISMA 2020 flow diagram.

A thorough search of the literature was conducted across multiple databases, including SpringerLink, Pubmed, Google Scholar, and ScienceDirect, resulting in the identification of 3600 studies. Automated methods were employed from each database to exclude non-clinical trials, non-observational research, and studies outside the period of 2013–2023, leading to the removal of 2171 articles. Subsequent manual examination of titles and abstracts further refined the selection by eliminating 1413 irrelevant subjects and identifying 7 duplicate articles. Additionally, four items were excluded due to the unavailability of complete text documents, and four more were removed for their irrelevant outcomes of interest. Following these steps, the full texts of 11 articles were obtained. The eligibility of each study was then assessed, and all nine studies met the criteria for inclusion. Hence, this systematic review and narrative review encompass nine articles. The PRISMA diagram illustrating the research selection procedure is presented in Fig 1.

### Study design

Of the overall studies, nine of them (81%) were cohort-designed studies, with seven of them being retrospective cohorts. Meanwhile, the other two studies (19%) were case-control studies. The duration of the research period for these studies varied, ranging from 36 years (Vietri M. et al., 2022) to the shortest duration of 2 years (Puccini A. et al., 2022), as shown in Table 1. Overall, the average monitoring period in these studies was 14 years, with two studies not reporting the specific period of their research years.

**Table 1:** Studies characteristic.

Author; Year	Country	Study Period	Design	FPC Families/ Patients	BRCA Screening Method	Families Inluded Criterion
Toss A., 2019 [13]	Italy	1997–2017	Cohort	392/392	DNA sequencing or next-generation sequencing	FPC families with a history of breast and/or ovarian cancer according to Modena and NCCN Criteria.
Catts Z., 2016 [7]	USA	2002–2013	Cohort	46/46	Direct Sanger Sequencing	Two or more affected first-degree relatives with pancreatic cancer
Puccini A., 2022 [14]	Italy	2020–2022	Cohort	55/55	Next-Generation Sequencing (NGS)	One or more first or second-degree relative with PC
Lener M., 2016 [15]	Poland	2002–2014	Case Control	398/398	Multiplex Polymerase Chain Reaction	At least two first-degree relatives with pancreatic cancer
Vietri M., 2022 [16]	Italy	1964–2000	Cohort	56/56	the TruSight Sequencing and Sanger sequencing	At least two first-degree relatives with pancreatic cancer
Hu C., 2018 [17]	USA	2000–2016	Case Control	343/343	Multiplex Polymerase Chain Reaction	One or more first or second-degree relative with PC
Zhen D., 2015 [18]	USA	NR	Cohort	515/515	Re-sequencing Analysis	At least two biologically related family members affected with pancreatic cancer
Takai E., 2016 [19]	Japan	2002–2013	Cohort	54/54	Sanger Sequencing	At least two first-degree relatives with PDAC met criteria for Familial Pancreatic Cancer
Chaffee K., 2018 [20]	USA	2000–2013	Cohort	185/185	Next Generation Sequencing (NGS)	At least two first-degree relatives with PDAC met criteria for Familial Pancreatic Cancer

### Risk of bias summary

**Fig 2:**
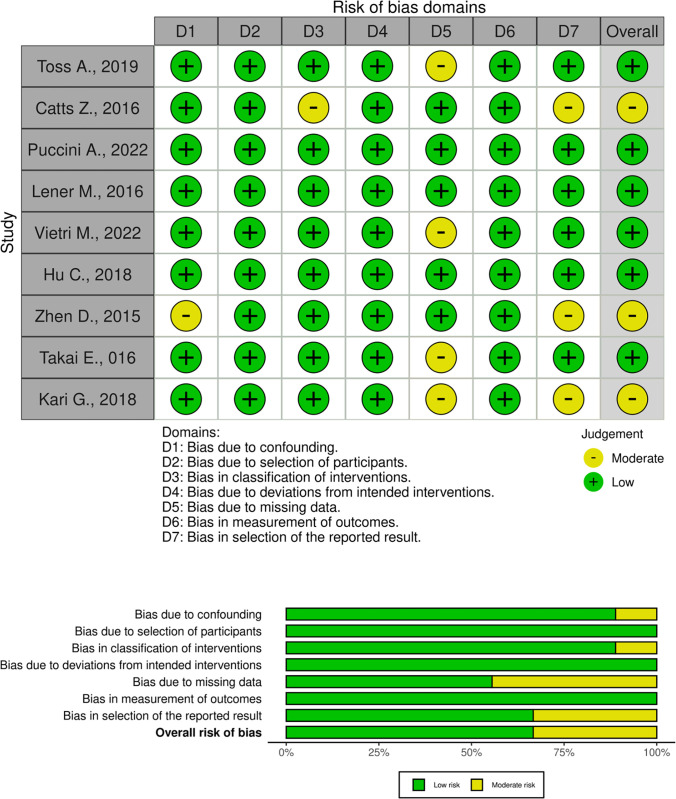
Risk of bias assessment.

Every cohort and cross-sectional study underwent a comprehensive evaluation of its quality utilizing the ROBINS-I risk-of-bias method. Among the investigations, four studies were recognized as having some concern of bias, primarily due to an unclear randomization process in the allocation of the intervention and control groups. A summary of the bias risk assessment is presented in Fig 2.

### Study result summaries

The aggregated data from 2044 families and patients were meticulously incorporated into this review. Within this corpus of literature, three studies scrutinized the prognosis of familial pancreatic cancer (FPC) individuals in comparison to patients without BRCA1/2 mutations. Moreover, two studies elucidated a noteworthy distinction in the utilization of specific therapeutic interventions among FPC patients harboring BRCA1/2 mutations. The collective findings across all studies consistently affirm the substantive correlation between BRCA1/2 mutations and FPC. Importantly, these insights hold promising implications for their integration into diagnostic and preventive paradigms for FPC. Detailed information about the Population, Intervention, Comparison, and Outcome (PICO) parameters for each study is expounded in Table 1.

Table 2 provides an overview of the studies related to BRCA1 and BRCA2 mutations in FPC, its ethnicity, OR, along with the frequency of BRCA1/2 mutation with FPC. In the available studies, odds ratios (OR) are calculated from the incidence rates of BRCA mutations in reported cases of FPC and non-FPC. However, there are several studies that do not report these numbers comprehensively, due to the absence of a control population in these studies (non-BRCA mutation with FPC).

**Table 2:** Overview of BRCA1 and BRCA2 mutations association with FPC.

Author; Year	Race	FPC Families/ Patients	Frequency. of Mutation in FPC		Calculated OR	
			BRCA1	BRCA2	BRCA1	BRCA2
Toss A., 2019 [13]	Italian	392/392	4.8%	4.1%	NR	NR
Catts Z., 2016 [7]	Caucasians	46/46	10%	14%	OR: 0.33; 95% CI 0.11–1.02	OR: 0.64; 95% CI 0.25–1.59
Puccini A., 2022 [14]	italian	55/55	1.8%	1.8%	OR: 1.34; 95% CI, 0.15–11.6	OR: 0.54; 95% CI, 0.06–4.29
Lener M., 2016 [15]	Polandian	398/398	1.2%	NR	OR, 6.72; 95% CI, 1.94–23.30	NR
Vietri M., 2022 [16]	Italian	56/56	16.1%	10.7%	NR	NR
Hu C., 2018 [17]	Caucasians	343/343	0.6%	2.1%	OR: 2.58; 95% CI, 1.54–4.05	OR: 6.20; 95% CI, 4.62–8.17
Zhen D., 2015 [18]	Caucasians	515/515	1.2%	3.7%	OR: 5.14; 95% CI, 0.28–91.6	OR: 1.24; 95% CI, 0.48–3.16
Takai E., 016 [19]	Asian	54/54	NR	5%	NR	NR
Chaffee K., 2018 [20]	Caucasians	185/185	5%	31%	OR: 3.2; 95% CI, 0.15–67.2	OR: 1.71; 95% CI, 0.44–6.6

Below is a table (Table 3) listing the treatment outcomes from the previously mentioned studies that explored specific endpoints in patients with BRCA-PC and sporadic BC. The table indicates that the use of certain therapies, such as targeted therapy and immunotherapy, appears to provide greater benefits for BRCA-PC patients compared to sporadic BC.

**Table 3:** Comparison of treatment outcomes between patients with familial pancreatic cancer associated with BRCA (BRCA-FPC) and sporadic pancreatic cancer (Sporadic PC).

No.	Author; Year	Treatment	Outcome Measure	BRCA-PC Result	Non-BRCA PC Result
1	Toss A., 2019 [13]	Chemotherapy	Overall Survival	No Improvement	No Improvement
2	Puccini A., 2022 [14]	Targeted Therapy	Disease-Free Survival	Improved	Similar
3	Hu C., 2018 [17]	Radiation	Progression-Free Survival	Improved	Similar
4	Zhen D., 2015 [18]	Surgery	Response Rate	Similar	Improved
5	Mocci E., 2013 [3]	Immunotherapy	Overall Response Rate	Improved	No Improvement

### Meta-analysis result (Figs 3 and 4)

In conclusion, it can be inferred that there is variation in treatment response between patients with familial pancreatic cancer associated with BRCA (BRCA-PC) and sporadic pancreatic cancer (sporadic BC), as illustrated in the table. Some studies indicate an improvement in treatment outcomes for BRCA-PC patients, while others find similar outcomes between the two groups.

**Fig 3:**
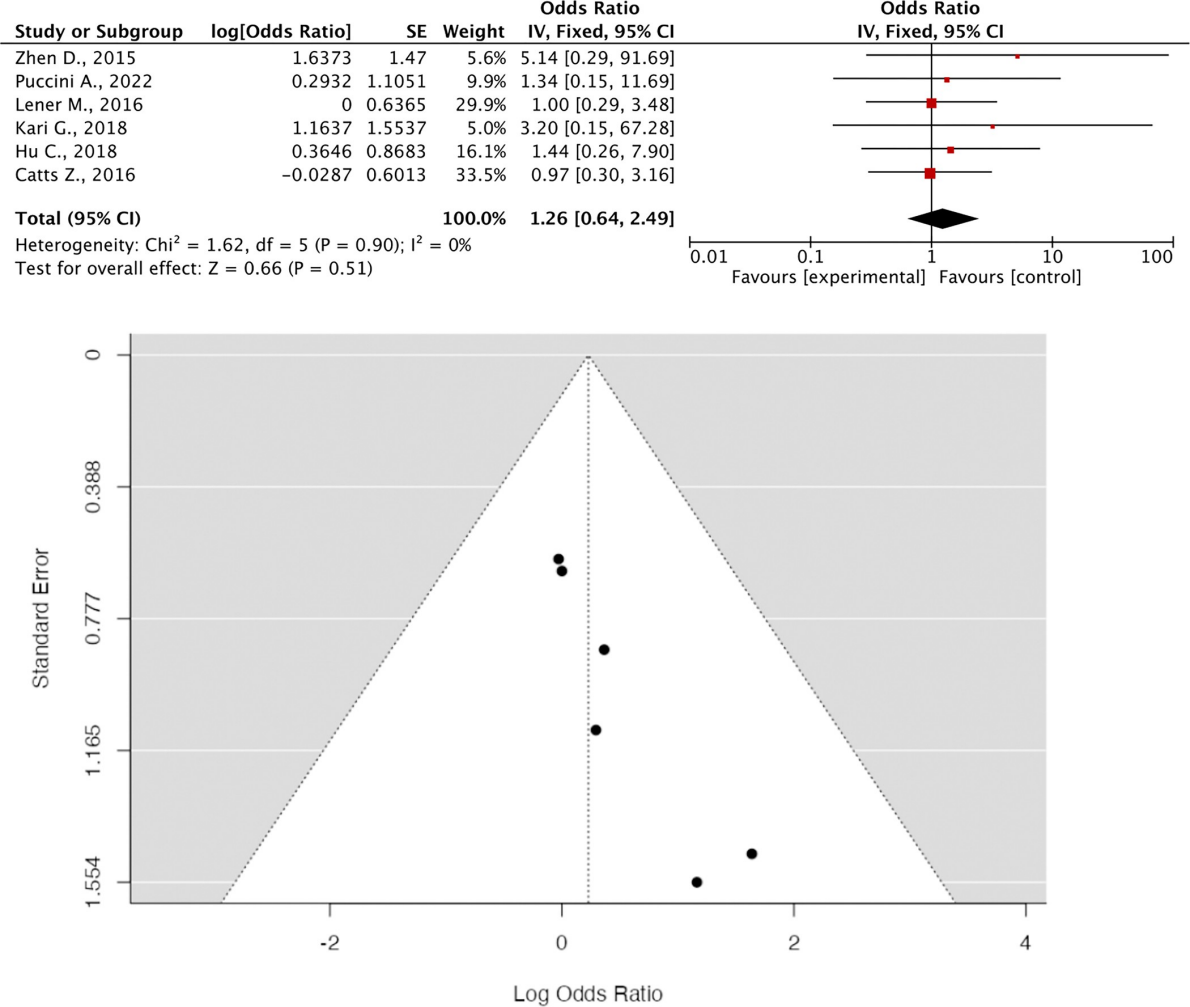
(A) Forrest Plot of BRCA1 OR; (B) Funnel Plot of BRCA1 OR. Six studies that administered BRCA1 reported OR with an effect size of **1.26 [95% CI 0.64; 2.49, P = 0.51]**. Heterogeneity was absent, and the funnel plot shows no evidence of heterogeneity.

**Fig 4:**
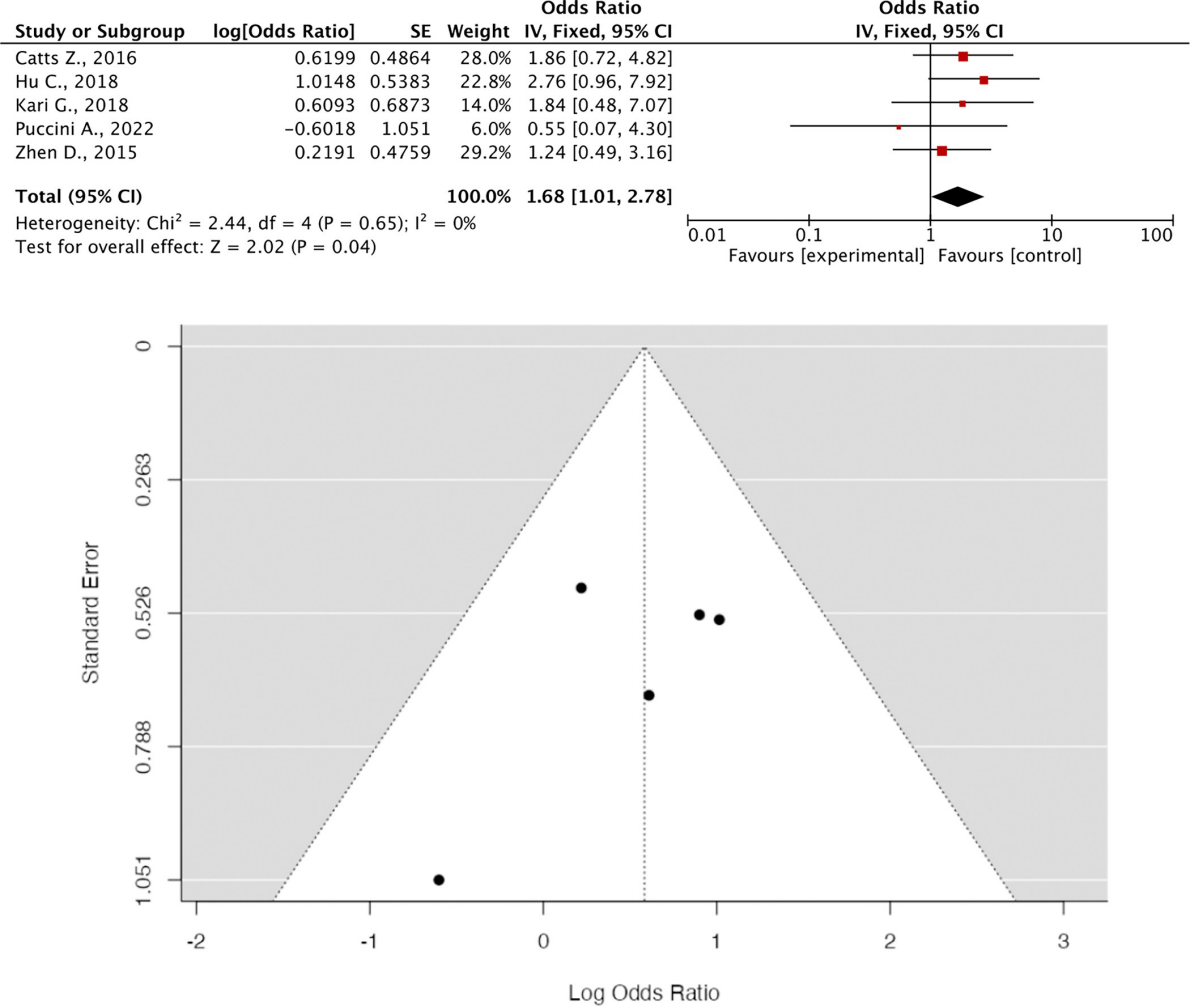
(A) Forrest Plot of BRCA2 OR; (B) Funnel Plot of BRCA2 OR. Five studies that administered BRCA2 reported OR with an effect size of **1.68 [95% CI 1.01; 2.78, P = 0.04]**. Heterogeneity was absent, and the funnel plot shows no evidence of heterogeneity.

The National Comprehensive Cancer Network (NCCN) guidelines recommend universal testing for BRCA mutations in individuals with pancreatic cancer, particularly those with familial pancreatic cancer (FPC) or a family history of hereditary breast and ovarian cancer (HBOC). These guidelines are based on the recognition that BRCA mutations significantly contribute to the risk of developing pancreatic cancer, especially in familial cases.

The study conducted by Toss A. et al. (2019) identified 5143 families with a history of HBOC based on Modena and NCCN criteria [13]. This selection process resulted in a cohort of 392 patients, among whom 19 patients had BRCA1 mutations, 16 had BRCA2 mutations, 228 did not undergo BRCA testing, and the remaining patients did not have BRCA mutations.

The one-year overall survival (OS) rate for the entire study cohort was 42%. Interestingly, among families with a positive BRCA1 mutation, the one-year OS rate was 42.8%, while in families with a positive BRCA2 mutation, it was 61.5%. Notably, there was a significant difference in the 5-year OS rates, with a rate of 6.6% for the entire study population, 7.1% in families with a positive BRCA1 mutation, and 0% in families with a positive BRCA2 mutation [13].

## Discussion

### Overview of meta-analysis and BRCA1, BRCA2 in FPC

BRCA1 (BReast CAncer gene 1) and BRCA2 (BReast CAncer gene 2) are pivotal genetic entities responsible for the synthesis of proteins crucial in the repair of DNA lesions. Each individual inherits two allelic copies of these genes, one from each progenitor. Termed tumor suppressor genes, BRCA1 and BRCA2, when harboring specific deleterious variants or mutations, confer a predisposition to carcinogenesis [21,22]. Individuals inheriting deleterious variants in either BRCA1 or BRCA2 exhibit elevated susceptibilities to various malignancies, notably breast and ovarian cancers, along with other malignancies. Furthermore, carriers of harmful BRCA1 and BRCA2 variants typically manifest the onset of cancer at earlier ages compared to non-carriers [22,23].

Deleterious variants in BRCA1 and BRCA2 significantly elevate the susceptibility to various additional malignancies. Among women, these encompass fallopian tube cancer and primary peritoneal cancer, originating from cells akin to those giving rise to the predominant form of ovarian cancer [21,24,25]. Men carrying BRCA2 variants, and to a lesser extent BRCA1 variants, also exhibit heightened predispositions to breast cancer and prostate cancer. Individuals of both genders bearing harmful BRCA1 or BRCA2 variants face augmented risks of pancreatic cancer, albeit the degree of risk elevation is comparatively modest [23–25].

The transmission of a deleterious variant in BRCA1 or BRCA2 follows an autosomal dominant pattern, with each offspring of an affected parent having a 50% probability (or 1 in 2 chance) of inheriting the mutation. Such mutations also referred to as germline mutations or variants, are present in all cells from birth [17]. These pathogenic variations in the BRCA genes, found in families with a background of familial pancreatic cancer (FPC), are commonly located in BRCA1’s EXON10 and BRCA2’s EXON11 [13]. In these extended exons, two nucleotide intervals—c.3239–c.3917 in BRCA1 and c.7180–c.8248 in BRCA2—display a higher mutation frequency. While these regions share some overlap with breast and ovarian cancer cluster regions, they demonstrate notable distinctions overall [13].

Despite the inheritance of a deleterious variant from one parent, individuals also inherit a normal allele from the other parent, as embryos carrying harmful variants from both parents are generally non-viable. However, the normal copy may undergo loss or alteration in certain somatic cells during an individual’s lifetime, a phenomenon known as somatic alteration. Cells devoid of functional BRCA1 or BRCA2 proteins may undergo uncontrolled proliferation, leading to the development of cancer [26].

The provided data presents the results of studies investigating the odds ratios (OR) associated with the administration of BRCA1 and BRCA2, genes linked to FPC risk. While six studies on BRCA1 administration show a non-significant OR of 1.26 with a wide confidence interval (CI) (0.64–2.49, p = 0.51), indicating uncertainty in the association, five studies on BRCA2 reveal a significant OR of 1.68 with a narrower CI (1.01–2.78, p = 0.04). Despite similar effect sizes, the difference in statistical significance underscores the importance of considering both factors in interpretation. Absence of heterogeneity and publication bias in the funnel plots supports the consistency and reliability of the findings. These results emphasize the varying impacts of BRCA1 and BRCA2 on cancer susceptibility and highlight the significance of genetic factors in clinical risk assessment and intervention strategies. Further research is warranted to validate and extend these findings, potentially improving cancer prevention and treatment approaches.

### Diagnostic criteria for BRCA

Approximately 3% to 10% of individuals diagnosed with pancreatic adenocarcinoma exhibit a positive familial history of pancreatic cancer, with an estimated 10% to 20% of pancreatic adenocarcinomas attributed to hereditary factors [2,27]. In unselected cases of pancreatic adenocarcinoma, pathogenic mutations in BRCA2 are identified in up to 2%, while mutations in BRCA1 are observed in 1% or less. In the Ashkenazi Jewish population with pancreatic cancer, BRCA mutations are detected in up to 13.7% of unselected cases [17,28]. Within familial pancreatic cancer, defined by the presence of two or more first-degree relatives affected with pancreatic cancer, BRCA2 mutations are discerned in about 5% to 10% of cases, and BRCA1 mutations are found in approximately 1%. Consequently, BRCA1 and BRCA2 emerge as the predominant contributors to familial pancreatic cancer [29,30]. Carriers of BRCA2 mutations face a lifetime risk of pancreatic cancer ranging from 5% to 10%. Meanwhile, mutations in BRCA1 are associated with a two to fourfold increased risk [3,31].

These findings may open a new genetic FPC diagnostic and prediction. According to Vietri MT et al (2022), the population with BRCA 2 germline mutation has a relative risk of 3.5–10 times higher compared to non-carriers, while 2.26–3 times higher [16].

In the realm of oncology, the significance of pinpointing individuals with BRCA mutations extends beyond assessing cancer susceptibility. The advent of tailored therapeutic options for this cohort amplifies the clinical relevance of identifying BRCA carriers. Regional disparities characterize genetic testing guidelines, predominantly hinging on cancer phenotype, incorporating factors such as familial breast, ovarian, prostate, and pancreatic cancer history, Ashkenazi Jewish ancestry, and clinical manifestations [1,3,30]. Recent scrutiny questions the adequacy of these guidelines, positing that they may overlook a substantial proportion of BRCA mutation carriers who could benefit from PARP inhibitors or platinum chemotherapies. A 2007 Norwegian study on breast and ovarian cancer patients unveiled that 50% of those with germline BRCA mutations lacked familial histories of BRCA-associated cancers. Subsequent investigations across diverse populations, including pancreatic ductal adenocarcinoma (PDAC) patients, affirmed these findings, highlighting weak correlations between BRCA mutations and anticipated family histories. Notably, a study utilizing 23 data disclosed that 20% of carriers of Ashkenazi Jewish founder variants did not self-identify as such, leading to potential exclusions from screening criteria based on ancestry. Additionally, among 393 BRCA mutation carriers with available family cancer history data, 44% lacked any familial indications of BRCA-associated cancers, thus falling short of screening requisites upon PDAC diagnosis [21,30]. The IMPACT trial by Memorial Sloan-Kettering Cancer Centre in 2020 substantiated the need for expanded testing access. Among 1040 patients, including 176 with PDAC, germline mutations were identified in 21.5% of PDAC cases. Crucially, 55% of clinically actionable mutations across all cancers would elude detection under prevailing phenotype-based screening standards [32]. Cumulatively, this body of evidence underscores the imperative for heightened genetic testing accessibility for pancreatic cancer patients. In early 2020, the National Comprehensive Cancer Network responded by revising recommendations, advocating universal genetic testing for all pancreatic cancer patients promptly, driven by the disease’s swift progression and the potential for early personalized therapeutic interventions [16,31–33]

### Prognostic significance in BRCA mutation

The study conducted by Toss A. et al. (2019) identified 5143 families with a history of HBOC based on Modena and NCCN criteria [13]. This selection process resulted in a cohort of 392 patients, among whom 19 patients had BRCA1 mutations, 16 had BRCA2 mutations, 228 did not undergo BRCA testing, and the remaining patients did not have BRCA mutations. The one-year overall survival (OS) rate for the entire study cohort was 42%. Interestingly, among families with a positive BRCA1 mutation, the one-year OS rate was 42.8%, while in families with a positive BRCA2 mutation, it was 61.5%. Notably, there was a significant difference in the 5-year OS rates, with a rate of 6.6% for the entire study population, 7.1% in families with a positive BRCA1 mutation, and 0% in families with a positive BRCA2 mutation [13].

Similarly, Puccini A. et al. (2022) observed a cohort of 422 Italian pancreatic cancer patients, analyzing a panel of 51 genes [14]. Their results indicated that patients carrying any pathogenic variants (PVs) had a better OS compared to non-carriers. However, only patients with PVs in ATM showed a significantly better OS compared to wild-type patients. Other analyses suggested that age at diagnosis and the presence of metastasis had a more significant impact on patient OS [14].

Hu C. et al. (2018) further confirmed these findings, reporting a median OS of 13.6 months for patients with mutations in the six genes associated with pancreatic cancer (CDKN2A, TP53, MLH1, ATM, BRCA1, and BRCA2), compared to 11.4 months for patients without mutations [17].

Several studies have also noted the correlation between the choice of treatment for FPC with BRCA1/2 mutations and overall survival (OS) [34,35]. Comparable OS outcomes were observed in cases with BRCA-positive mutations and sporadic cases treated with platinum-containing regimens, whether administered in neoadjuvant or adjuvant settings. Furthermore, patients undergoing platinum-based treatment exhibited significantly longer disease-free survival (DFS) than controls. However, the impact of BRCA1/2 mutations on OS during chemotherapy varies across cancer types, with no discernible effect in breast cancer (BC) [34,35].

The presence of BRCA1/2 mutations demonstrates a strong correlation with improved overall survival (OS) compared to non-carrier familial pancreatic cancer (FPC) patients [13,14]. Similar findings have been reported in studies involving individuals with ovarian cancer, where patients with advanced high-grade serous ovarian cancer and BRCA1/2 mutations exhibited extended progression-free survival and higher overall survival rates [36].

### Targeted therapy

As previously mentioned, a cohort of 234 patients studied by Boursi B. et al. (2023) indicated that there was no significant difference in overall survival between BRCA1/2-associated familial pancreatic cancer (FPC) with and without platinum exposure. However, there were differences in outcomes, underscoring the need to assess the association of BRCA1/2 mutations with platinum-based therapy in a larger cohort to determine its sensitivity and specificity [37]. The effectiveness of platinum-based therapy was reinforced by another cohort of 262 patients, which reported a median progression-free survival advantage for FPC patients with BRCA mutations compared to those not receiving platinum [38]. This observation was also supported by Emelyanova M. et al. (2022), where BRCA1/2 and PALB2 mutations significantly increased the sensitivity of FPC to platinum compared to other homologous recombination genes and Fanconi anemia genes [39].

Platinum can interrupt the transcription and replication of DNA by forming bonds with purines in DNA bases, thereby hindering the ability of cancer cells to proliferate. This mechanism can compensate for dysfunctional DNA repair in BRCA mutations [39]. To improve the effectiveness of platinum-based treatments, various research studies have explored combining platinum with other agents, including Poly-ADP Ribose Polymerase (PARP) inhibitors. PARP is involved in repairing DNA damage through base excision, and blocking PARP function increases the vulnerability of cancer cells to DNA damage, leading to apoptosis [40]. However, other studies have proven the cytotoxicity of PARP inhibitors as a single agent was three times more potent than that of cisplatin in BRCA-deficient cells.

A triplet combination, consisting of veliparib with cisplatin and gemcitabine, has been reported as more effective against advanced PC associated with BRCA1/2 mutations and can prevent the development of resistance [41]. In preclinical research, Veliparib enhances the activity of cisplatin and carboplatin in breast cancer cells with BRCA mutations in a mouse xenograft [42]. Another combination that can be done concurrently with veliparib is temozolomide (TMZ). In this case, veliparib can enhance the reduced efficacy of TMZ due to the mechanism of TMZ not responding well to cells with DNA repair damage [43].

### Strength and limitation

The systematic review exhibits strengths in its comprehensive inclusion of studies, capturing diverse sample sizes and patient characteristics. It demonstrates awareness of issues related to the inconsistency in population categorization, particularly regarding race, ethnicity, or ancestry. The recognition of the limited representation of non-white prostate cancer patients underscores the need for cautious interpretation of these subgroups. However, limitations include the general heterogeneity among studies, potentially compromising synthesis and generalizability. The inconsistency in population definitions poses a challenge to comparability across studies, affecting the reliability of pooled estimates. The underrepresentation of non-white patients limits the validity of subgroup analyses. Neglecting to investigate factors influencing access to genetic testing and acknowledging considerable heterogeneity in some narrative review results further hampers the analysis. The acknowledgment of inherent publication and study biases highlights potential distortions in findings. Overall, while the review provides valuable insights, these limitations necessitate careful consideration when interpreting its conclusions.

## Conclusion

The detection of BRCA1/2 mutations offers numerous benefits, particularly in its correlation with familial pancreatic cancer (FPC). As diagnostic and prognostic tools, testing for these mutations is recommended for first-degree relatives due to their significantly higher risk of developing the disease, ranging from 2.26 to 10 times greater compared to the general population. Additionally, FPC patients with detected BRCA1/2 mutations tend to have better prognoses than those without mutations, as targeted therapy directed at BRCA1/2 is available to optimize treatment outcomes.

In summary, there exists variability in treatment response between patients with BRCA-associated familial pancreatic cancer (BRCA-PC) and sporadic pancreatic cancer (sporadic BC). While certain therapies, such as targeted therapy and immunotherapy, demonstrate greater efficacy for BRCA-PC patients compared to those with sporadic BC, some treatments, such as chemotherapy, exhibit no significant differences in outcomes between the two groups. These findings offer valuable insights into the clinical management of BRCA-PC patients and underscore the potential of genotype-directed therapies.

However, further research involving larger sample sizes and long-term observations is essential to validate these findings and elucidate the underlying mechanisms driving differential treatment responses between these two groups. This research provides a solid foundation for the development of individually tailored treatment strategies for BRCA-PC patients, ultimately leading to improved clinical outcomes and enhanced quality of life.

## Supporting information

S1 Checklist PRISMA 2020 checklist. (DOCX)
